# Outcomes with guideline-directed medical therapy and cardiac implantable electronic device therapies for patients with heart failure with reduced ejection fraction

**DOI:** 10.1016/j.hroo.2024.01.004

**Published:** 2024-01-24

**Authors:** John L. Mignone, Kevin M. Alexander, Michael Dobbles, Kyle Eberst, Gregg C. Fonarow, Kenneth A. Ellenbogen

**Affiliations:** ∗ Division of Cardiology, Swedish Medical Center, Seattle, Washington; † Division of Cardiovascular Medicine, Department of Medicine, Stanford University, Stanford, California; ‡ egnite, Inc., Aliso Viejo, California; § Ahmanson–UCLA Cardiomyopathy Center, University of California, Los Angeles, Los Angeles, California; || Department of Cardiology, Virginia Commonwealth School of Medicine, Richmond, Virginia

**Keywords:** Heart failure with reduced ejection fraction, Guideline-directed medical therapy, Implantable cardioverter-defibrillators, Cardiac resynchronization therapy, Therapy optimization

## Abstract

**Background:**

Limited real-world evidence exists for outcomes with contemporary guideline-directed medical therapy (GDMT) or GDMT with implantable cardioverter-defibrillator (ICD)/cardiac resynchronization therapy defibrillator (CRT-D) therapy for patients with heart failure with reduced ejection fraction (HFrEF) and left ventricular ejection fraction (LVEF) ≤35%.

**Objective:**

The present study aimed to assess survival associated with GDMT or GDMT with ICD/CRT-D therapy.

**Methods:**

This retrospective observational study included real-world de-identified data from January 1, 2016, to December 19, 2023, from 24 U.S. institutions per participating institutional agreements (egnite Database; egnite, Inc.). Patients with a diagnosis of HFrEF and an echocardiographic study documenting LVEF ≤35% were included for analysis.

**Results:**

Of 43,591 patients with eligible index event of LVEF ≤35%, prescription history through ≥1 year preindex, and no ICD/CRT-D therapy preindex, mean ± standard deviation age at index was 71.2 ± 13.2 years; 14,805 (34.0%) patients were female. At 24 months, an estimated 99.1% (95% confidence interval [CI] 99.0%–99.2%), 89.9% (95% CI 89.7%–90.1%), 54.8% (95% CI 54.4%–55.2%), and 17.2% (95% CI 16.9%–17.5%), had ≥1, 2, 3, or all 4 GDMT classes prescribed, respectively; an estimated 15.7% (95% CI 15.3%–16.1%) had device placement. Of those without a device, by 24 months, an estimated 45.1% (95% CI 44.4%–45.7%) had a documented LVEF >35%. Counts of GDMT classes prescribed as well as ICD/CRT-D device therapy were associated with lower mortality risk in this population, even after adjustment for patient age, sex, and comorbidities.

**Conclusion:**

Both GDMT classes prescribed and device therapy were independently associated with lower mortality risk, even in the presence of more GDMT options for this more contemporary population.

**Key Findings**

▪ Both greater prescription of guideline-directed medical therapy (GDMT) classes and implantable cardioverter-defibrillator/cardiac resynchronization therapy defibrillator device therapy were associated with greater survival in this patient population.
▪ A general trend toward greater adoption of at least 3 classes of GDMT was observed in this contemporary dataset, although room for improvement remains.
▪ These findings not only demonstrate a real-world survival benefit associated with contemporary GDMT for patients with heart failure with reduced ejection fraction, but also suggest an incremental independent survival benefit associated with implantable cardioverter-defibrillator/cardiac resynchronization therapy defibrillator therapy, even in an era with greater options in GDMT.

## Introduction

The past decade has yielded numerous promising advances in treatment options for patients with heart failure withreduced ejection fraction (HFrEF)—perhaps most notably in guideline-directed medical therapy (GDMT), in whichmultiple randomized clinical trials resulted in in U.S. Food and Drug Administration approvals of the angiotensinreceptor–neprilysin inhibitor (ARNI) sacubitril/valsartan^1^ and subsequently of the sodium-glucose cotransporter 2 inhibitors (SGLT2I) empagliflozin^2^ and dapagliflozin^3^ for this patient population. This proliferation of GDMT options in HFrEF is now recognized in recent guidelines for the management of heart failure.^4^ Several studies have since reported on resultant practice patterns or care models^5^^,^^6^ as well as on potential benefits^6^, ^7^, ^8^, ^9^ associated with more contemporary GDMT.

However, because many pivotal trials for device-based therapies for HFrEF, including implantable cardioverter-defibrillators (ICDs)^10^^,^^11^ and cardiac resynchronization therapy defibrillators (CRT-Ds),^12^ were completed in the years prior to these advances, limited evidence exists for treatment patterns and patient outcomes associated with both contemporary GDMT and ICD/CRT-D therapy for patients who meet guideline recommendations for both treatment options (including left ventricular ejection fraction [LVEF] ≤35%). The present study sought to address this evidence gap using a large multicenter real-world database.

## Methods

### Data source

We conducted a retrospective multicenter cohort study using a de-identified dataset with data capture between January 1, 2016, and December 19, 2023, from the egnite Database (egnite, Inc., Aliso Viejo, CA), which was developed per participating institutional agreements. A study exemption from institutional review board oversight was obtained from the WCG IRB Group (Puyallup, WA). All de-identified datasets used were compliant with the Health Insurance Portability and Accountability Act.

The egnite Database consists of data derived from the CardioCare platform (egnite, Inc.), which is a digital health platform used by institutions across the United States to help manage their cardiovascular patient population. At the time of the present study, data from 24 institutions were included in the Database with appropriate permissions.

Data were prepared for the present study following initial data quality assessments by a clinical team. The prespecified master study dataset was generated from the egnite Database by consolidating de-identified data contributed by individual institutions within the dates specified (January 1, 2016, to December 19, 2023). De-identified patient data made available for extraction for this study dataset included: age (with ages 90 years or greater truncated to a single category of ≥90 years for irreversible deidentification); sex (male, female, unknown); echocardiographic report data; medication prescriptions and date of prescription entry; device-related procedure events (including dates of procedures); diagnosed comorbidities (as defined in Supplemental Table 1); and documented date of death per institutional records, if applicable. Device placement was identified via eligible procedure (*International Classification of* *Diseases–Tenth* *Revision* or Current Procedural Terminology) codes. Comorbidities were generally identified per eligible *International Classification of* *Diseases–Tenth* *Revision* codes for each diagnosis. Dosing and names of individual medications are documented in the egnite Database, but the present study prespecified a step to consolidate at the level of GDMT classes as part of the irreversible dataset extraction plan.

### Study design

Key inclusion criteria for this study included a diagnosis of heart failure; availability of ≥1 echocardiographic report post–heart failure diagnosis with documented LVEF ≤35%, with the first such report after diagnosis of heart failure used as the study index event; and prescription history through ≥1 year preindex. Exclusion criteria included documented treatment with an ICD/CRT-D preindex. For purposes of this study, GDMT included all 4 classes of foundational medications with established survival benefit,^4^ including an angiotensin-converting enzyme inhibitor (ACEI)/angiotensin receptor blocker (ARB)/ARNI, beta-blocker, mineralocorticoid receptor antagonist, and SGLT2I (given that SGLT2I were commercially available prior to their incorporation into the most recent guideline document andpatients could thus have been treated with, and benefited from, them throughout the study window). The primary outcome of interest in this study was all-cause mortality.

### Statistical analysis

Descriptive summary statistics of patient characteristics were reported either as number and percentage for categorical variables or as mean ± SD or median (interquartile range) for continuous variables, as appropriate. Analyses of treatment rates, LVEF improvement to >35%, and all-cause mortality were examined using Kaplan-Meier analyses, with first documented echocardiogram with LVEF ≤35% as the index event. Mortality analysis used prescribed GDMT class count and device placement as categorical time-varying covariates and censored patients at last clinical encounter; treatment rate and LVEF improvement analysis censored patients at last clinical encounter/death. A multivariable Cox regression model was used to further assess the association between GDMT classes prescribed and device therapy with the outcome of interest after adjustment for patient age, sex, and comorbidities.

All analyses were conducted using Databricks 13.3 LTS (Databricks, San Francisco, CA; Apache Spark 3.4.1 (Apache Software Foundation, Wilmington, DE); Scala 2.12 (École Polytechnique Fédérale Lausanne, Lausanne, Switzerland); R version 4.2.2 (R Foundation for Statistical Computing, Vienna, Austria); R survival package 3.5.3 (https://www.rdocumentation.org/packages/survival/versions/3.5-7).

## Results

**Figure 1:**
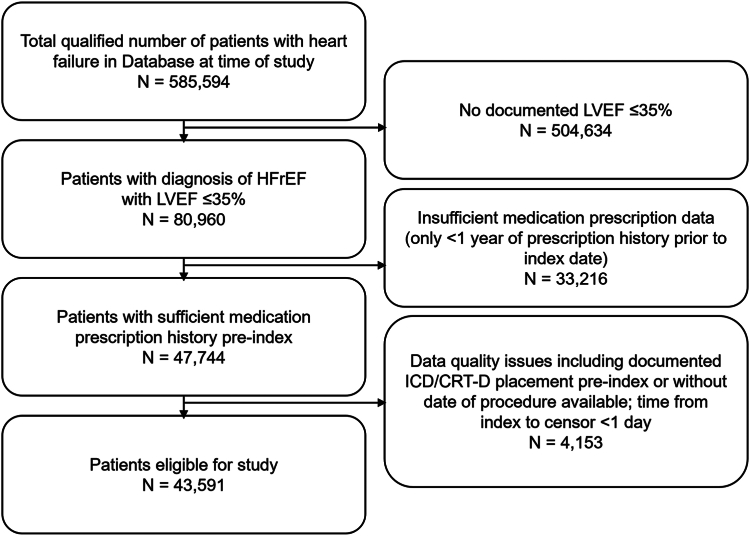
Flowchart of participant selection. CRT-D = cardiac resynchronization therapy defibrillator; HFrEF = heart failure with reduced ejection fraction; ICD = implantable cardioverter-defibrillator; LVEF = left ventricular ejection fraction.

A total of 43,591 patients were included for study analyses with a diagnosis of heart failure and an eligible documented LVEF ≤35%, prescription history through ≥1 year preindex, and no device therapy preindex (see flowchart summary of patient selection provided [Figure 1]). Patient characteristics are summarized in Table 1. Of the included patients, mean ± standard deviation age at index was 71.2 ± 13.2 years, 14,805 (34.0%) were female, and overall median time from index to censor/death was 13.8 (interquartile range: 3.9–28.4) months.

**TABLE 1:** Patient demographics and key clinical characteristics

	Overall	0-class GDMT	0-class GDMT + device	1-class GDMT	1-class GDMT + device	2-class GDMT	2-class GDMT + device	3-class GDMT	3-class GDMT + device	4-class GDMT	4-class GDMT + device
Number (% of overall)	43,591 (100.0)	627 (1.4)	13 (<0.1)	3,584 (8.2)	135 (0.3)	13,543 (31.1)	1,052 (2.4)	12,315 (28.3)	1,878 (4.3)	8,405 (19.3)	2,039 (4.7)
Age, y	71.2 ± 13.2	72.3 ± 16.0	72.7 ± 11.6	75.6 ± 12.5	74.8 ± 11.5	73.6 ± 13.0	72.2 ± 10.9	71.2 ± 12.8	70.3 ± 11.3	66.6 ± 13.2	66.0 ± 12.1
Key clinical history/comorbidities											
Diabetes	19,430 (44.6)	184 (29.3)	4 (30.8)	1,390 (38.8)	53 (39.3)	5,857 (43.2)	414 (39.4)	5545 (45.0)	791 (42.1)	4,198 (49.9)	994 (48.7)
CAD	30,627 (70.3)	374 (59.6)	8 (61.5)	2,434 (67.9)	97 (71.9)	9,637 (71.2)	799 (76.0)	8738 (71.0)	1390 (74.0)	5,730 (68.2)	1,420 (69.6)
MI	11,255 (25.8)	132 (21.1)	4 (30.8)	947 (26.4)	21 (15.6)	3,731 (27.5)	235 (22.3)	3260 (26.5)	425 (22.6)	2,023 (24.1)	477 (23.4)
Stroke	6,386 (14.6)	85 (13.6)	1 (7.7)	575 (16.0)	17 (12.6)	2,233 (16.5)	133 (12.6)	1827 (14.8)	221 (11.8)	1,038 (12.3)	256 (12.6)
COPD	9,745 (22.4)	145 (23.1)	1 (7.7)	853 (23.8)	36 (26.7)	3,199 (23.6)	225 (21.4)	2907 (23.6)	365 (19.4)	1,636 (19.5)	378 (18.5)
Renal disease	16,961 (38.9)	203 (32.4)	4 (30.8)	1,524 (42.5)	58 (43.0)	6,184 (45.7)	438 (41.6)	4648 (37.7)	685 (36.5)	2,667 (31.7)	550 (27.0)
Metastatic cancer	1,345 (3.1)	46 (7.3)	1 (7.7)	168 (4.7)	1 (0.7)	497 (3.7)	18 (1.7)	381 (3.1)	33 (1.8)	185 (2.2)	15 (0.7)
GDMT class prescribed											
ACEI/ARB/ARNI	40,677 (93.3)	0 (0.0)	0 (0.0)	2,159 (60.2)	61 (45.2)	12,972 (95.8)	1,002 (95.2)	12,180 (98.9)	1,859 (99.0)	8,405 (100.0)	2,039 (100.0)
Beta-blocker	39,478 (90.6)	0 (0.0)	0 (0.0)	1,274 (35.5)	69 (51.1)	12,745 (94.1)	996 (94.7)	12,108 (98.3)	1,842 (98.1)	8,405 (100.0)	2,039 (100.0)
MRA	21,914 (50.3)	0 (0.0)	0 (0.0)	110 (3.1)	3 (2.2)	948 (7.0)	68 (6.5)	8,978 (72.9)	1,363 (72.6)	8,405 (100.0)	2,039 (100.0)
SGLT2I	15,195 (34.9)	0 (0.0)	0 (0.0)	41 (1.1)	2 (1.5)	421 (3.1)	38 (3.6)	3,679 (29.9)	570 (30.4)	8,405 (100.0)	2,039 (100.0)

Values are n (%) or mean ± SD. Device specifically refers to placement of an ICD/CRT-D device.

ACEI = angiotensin-converting enzyme inhibitor; ARB = angiotensin receptor blocker; ARNI = angiotensin receptor–neprilysin inhibitor; CAD = coronary artery disease; COPD = chronic obstructive pulmonary disease; CRT-D = cardiac resynchronization therapy defibrillator; GDMT = guideline-directed medical therapy; ICD = implantable cardioverter-defibrillator; MI = myocardial infarction; MRA = mineralocorticoid receptor antagonist; SGLT2I = sodium-glucose cotransporter 2 inhibitor.

At index, 96.3%, 79.3%, 36.8%, and 8.8% of patients had ≥1, 2, 3, or all 4 GDMT classes prescribed, respectively. In contrast, at 24 months postindex, an estimated 99.1% (95% confidence interval [CI] 99.0%–99.2%), 89.9% (95% CI 89.7%–90.1%), 54.8% (95% CI 54.4%–55.2%), and 17.2% (95% CI 16.9%–17.5%) had ≥1, 2, 3, or all 4 GDMT classes prescribed, respectively, and 15.7% (15.3%–16.1%) had device placement. Of patients without a device, by 24 months, an estimated (95% CI) 45.1% (95% CI 44.4%–45.7%) had a documented LVEF >35%.

**Figure 2:**
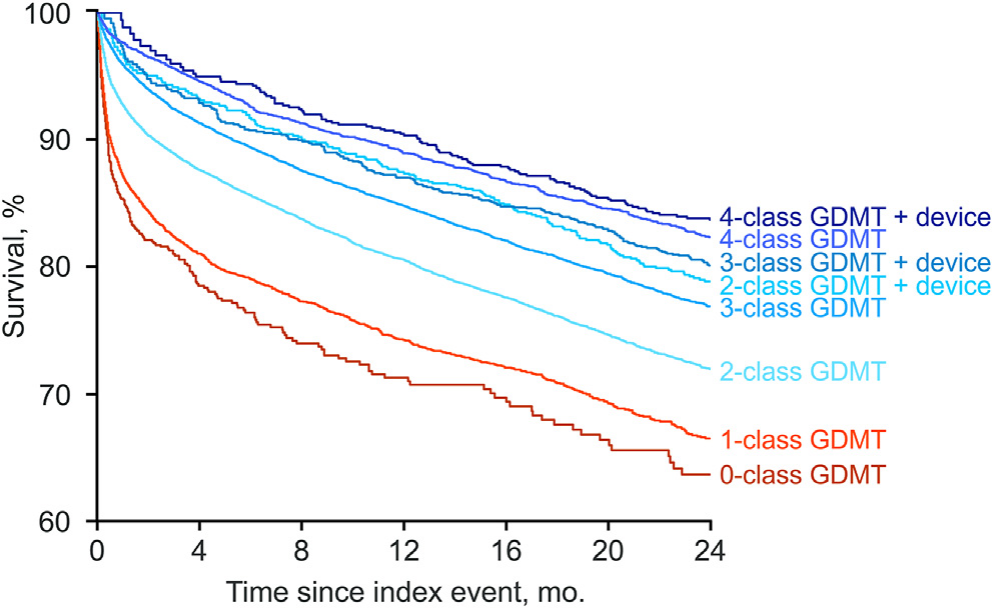
Kaplan-Meier curves for all-cause mortality by treatment modality. Device specifically refers to placement of an ICD/CRT-D device. CRT-D = cardiac resynchronization therapy defibrillator; GDMT = guideline-directed medical therapy; ICD = implantable cardiac defibrillator.

Cohorts without a device and 0, 1, 2, 3, or 4 GDMT classes prescribed exhibited an estimated 24-month survival rate of 63.7% (95% CI 59.5%–68.2%), 66.5% (95% CI 64.8%–68.3%), 72.0% (95% CI 71.1%–72.9%), 76.9% (95% CI 76.0%–77.8%), and 82.3% (95% CI 81.1%–83.6%), respectively (Figure 2). Cohorts with a device and 2, 3, or 4 GDMT classes prescribed had an estimated 24-month survival rate of 78.9% (95% CI 75.6%–82.3%), 80.1% (95% CI 77.3%–83.0%), and 83.7% (95% CI 80.4%–87.1%), respectively (Figure 2). There were relatively insufficient data for survival analysis of those with a device and 0 or 1 GDMT classes prescribed. For patients with 2-class GDMT, the most common combination of GDMT classes was an ACEI/ARB/ARNI + beta-blocker, while for patients with 3-class GDMT, the most common combination of GDMT classes prescribed was an ACEI/ARB/ARNI + beta-blocker + mineralocorticoid receptor antagonist (Supplemental Table 2). Of patients without a device, 53.8% were prescribed ≥3 GDMT classes vs 76.5% of those with a device; lower proportions were prescribed all 4 GDMT classes (21.8% vs 39.8%, respectively; Supplemental Table 2).

**Figure 3:**
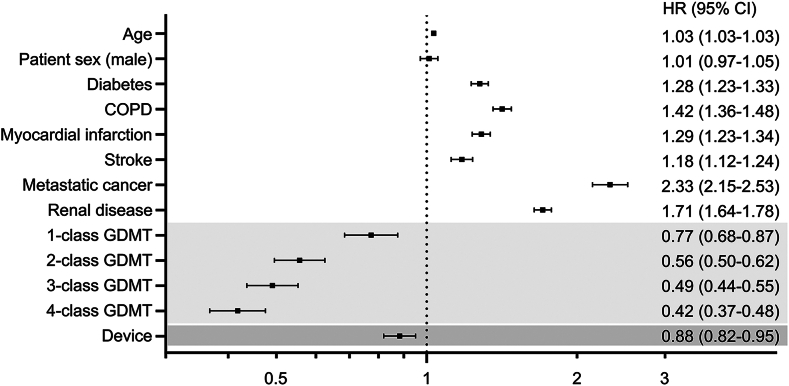
Modeled mortality risk by treatment modality. Hazard ratio for age expressed as per 1-year increase. Device specifically refers to placement of an ICD/CRT-D device. CI = confidence interval; COPD = chronic obstructive pulmonary disease; CRT-D = cardiac resynchronization therapy defibrillator; GDMT = guideline-directed medical therapy; HR = hazard ratio; ICD = implantable cardiac defibrillator.

In the multivariable analysis performed to account for observed imbalances in clinical characteristics across cohorts that may confound the present findings, after adjustment for patient age, sex, and comorbidities, GDMT was associated with a lower mortality risk compared with no GDMT, with a trend toward lower mortality risk with each additional GDMT class prescribed (Figure 3). In addition, ICD/CRT-D therapy was further independently associated with lower mortality risk in this population.

## Discussion

To our knowledge, the present study is the first multicenter real-world demonstration of incremental benefit associated with contemporary GDMT and ICD/CRT-D therapy in this patient population and represents an advance in the available evidence vs the historical pivotal studies for these device therapies. Specifically, in addition to demonstrating a real-world survival benefit associated with contemporary GDMT for patients with HFrEF, these data also suggest an incremental survival benefit associated with ICD/CRT-D therapy, even in an era with greater options for foundational GDMT.

The proportions of patients prescribed GDMT in this study and their associated outcomes are consistent with expectations per other reports describing suboptimal real-world implementation of GDMT for patients with HFrEF (eg, Change the Management of Patients with Heart Failure [CHAMP-HF] and, more recently, a Swedish Heart Failure Registry study and a population-based study in Alberta, Canada)^5^^,^^13^, ^14^, ^15^ despite well-established survival benefits conferred by these therapy options. Compared with those findings, we did observe increases in medication class prescribing patterns and a trend toward greater adoption of 3-class GDMT in this contemporary dataset, but arguably much room for improvement remains.

Finally, it is important to appreciate that the survival benefits associated with ICD/CRT-D therapy in this study were observed in a patient population with exposure to a more modern medical regimen, including even SGLT2I, and with 3 of 4 patients with a device also having been prescribed ≥3 GDMT classes. For perspective, the pivotal studies for these device-based therapies were conducted nearly 2 decades ago when fewer GDMT options were available. Specifically, baseline medical therapy consisted of just an ACEI/ARB and beta-blocker in the SCD-HeFT (Sudden Cardiac Death in Heart Failure Trial) trial^10^ and an ACEI/ARB, beta-blocker, and spironolactone in the COMPANION (Comparison of Medical Therapy, Pacing, and Defibrillation in Heart Failure) trial.^12^ Further study is needed to evaluate the present observed associations in a prospective manner, especially in patients prescribed maximal (4-class) GDMT who ultimately represented a relative minority of this study population.

### Strengths and limitations

Strengths of this study include the size, scope, recency, and generalizability of the dataset used, with opportunities for further analysis of treatment patterns and effects. Limitations of this study include possible variability due to regional differences in access to care and/or variability between programs, and data limited to patients who were selected in a clinical setting for an echocardiogram. Multivariable modeling was performed in an effort to better characterize the influence of GDMT and ICD/CRT-D therapy in this population, but it is important to recognize this carries notable limitations, including but not limited to the fact that there may be residual confounding due to unmeasured covariates that may influence the results. Even for all of those covariates that were available for analysis, as with any retrospective observational study, there is a possibility of missingness of data, and the same could be true for data on observed mortality events, which were site reported in this dataset. Taken together, the present work should be considered a hypothesis-generating study in which findings should not be interpreted as any estimates of true causal effect.

## Conclusion

The present study observed that both higher counts of GDMT classes prescribed and device therapy with GDMT were found to be associated with greater survival. Further study is needed to prospectively assess outcomes with specific contemporary GDMT regimens and ICD/CRT-D therapy for HFrEF.
