# Supplementary material for: Outcomes with guideline-directed medical therapy and cardiac implantable electronic device therapies for patients with heart failure with reduced ejection fraction
Source: Heart Rhythm O2. 2024 Jan 24;5(3):168–73. doi: 10.1016/j.hroo.2024.01.004 (PMC10980920; doi:10.1016/j.hroo.2024.01.004)
Supplement: Supplemental Material [file mmc1.docx]

**Supplemental Materials

Outcomes with Guideline-directed Medical Therapy and Cardiac Implantable Electronic Device Therapies for Patients with Heart Failure with Reduced Ejection Fraction**

John L. Mignone, MD, PhD^1^; Kevin M. Alexander, MD^2^; Michael Dobbles, MS^3^; Kyle Eberst, MBA^3^; Gregg C. Fonarow, MD^4^; Kenneth A. Ellenbogen, MD^5^

^1^Division of Cardiology, Swedish Medical Center, Seattle, WA, USA
^2^Division of Cardiovascular Medicine, Department of Medicine, Stanford University, Stanford, CA, USA
^3^egnite Inc., Aliso Viejo, CA, USA
^4^Ahmanson-University of California, Los Angeles Cardiomyopathy Center, University of California-Los Angeles, Los Angeles, CA, USA
^5^Department of Cardiology, Virginia Commonwealth School of Medicine, Richmond, VA, USA

**Supplemental Table** **1. Variable Definitions**

| Parameter | Definition(s) |
| --- | --- |
| Age | Extracted from EMR data (deidentified with ages 90 or greater truncated to a single category of ≥90 per Safe Harbor Methodology) |
| HFrEF | 'I50.2x', 'I50.4x', 'I50.82' OR 'I50.1x', 'I50.3x', 'I50.81x', 'I50.83', 'I50.84', 'I50.89', 'I50.9' AND subsequent echocardiogram with documented LVEF <40% |
| Metastatic cancer | 'C77.x', 'C78.x', 'C79.x' |
| CAD | ''I20.1', 'I20.2', 'I21.x', 'I22.x', 'I23.x', 'I24.x', 'I25.x', 'Z98.61' |
| COPD | 'J44.x' |
| Documented death | Expired patient status/date of death from institution |
| Diabetes | 'E10.x', 'E11.x', 'E13.x' |
| LVEF | Value extracted from echocardiographic procedure report |
| MI | 'I21.x', 'I22.x', 'I23.x' |
| Patient sex | Extracted from EMR data |
| Renal disease | 'N18.x’, ‘N19.x', 'Z91.15', 'Z99.2', 'I12.0', 'I12.9', 'I13.x' |
| Stroke | 'I63.x', 'Z86.73', 'G45.x' |
| ACEI / ARB / ARNI | Documented prescription of benazepril, captopril, enalapril, fosinopril, lisinopril, moexipril, perindopril, quinapril, ramipril, trandolapril, azilsartan medoxomil, candesartan cilexetil, irbesartan, losartan, olmesartan medoxomil, telmisartan, valsartan, or sacubitril/valsartan |
| Beta blocker | Documented prescription of bisoprolol, carvedilol, or metoprolol succinate |
| MRA | Documented prescription of eplerenone, spironolactone, or finerenone |
| SGLT2I | Documented prescription of dapagliflozin, empagliflozin, canagliflozin, or sotagliflozin |
| ICD / CRT-D placement | Procedure coded as 0JH608Z, 33240, 33249, 33262, 33270, A33249, A33270, 33230, 33263, 0JH638Z, 0JH808Z, 0JH838Z, 0JH609Z, 0JH639Z, 33264, 33249 + 33225, 33231, 0JH809Z, 0JH839Z |

ACEI = angiotensin-converting enzyme inhibitor; ARB = angiotensin receptor blocker; ARNi = angiotensin receptor-neprilysin inhibitor; CAD = coronary artery disease; COPD = chronic obstructive pulmonary disease; CRT-D = cardiac resynchronization therapy defibrillator; EMR = electronic medical record; HFrEF = heart failure with reduced ejection fraction; ICD = implantable cardiac defibrillator; LVEF = left ventricular ejection fraction; MI = myocardial infarction; MRA = mineralocorticoid receptor antagonist; SGLT2I = sodium glucose cotransporter-2 inhibitor.

**Supplemental Table 2. Comparisons of GDMT classes/combinations prescribed.**

|  | GDMT | | GDMT + device**^a^** | |
| --- | --- | --- | --- | --- |
| No. (%) | Overall (n = 38,474) | Within  class-count | Overall (n = 5,117) | Within  class-count |
| **0-class** | **627 (1.6)** | **N/A** | **13 (0.3)** | **N/A** |
| **1-class** | **3,584 (9.3)** | **N/A** | **135 (2.6)** | **N/A** |
| ACEI / ARB / ARNI | 2,159 (5.6) | 2,159 (60.2) | 61 (1.2) | 61 (45.2) |
| Beta blocker | 1,274 (3.3) | 1,274 (35.5) | 69 (1.3) | 69 (51.1) |
| MRA | 110 (0.3) | 110 (3.1) | 3 (0.1) | 3 (2.2) |
| SGLT2I | 41 (0.1) | 41 (1.1) | 2 (<0.1) | 2 (1.5) |
| **2-class** | **13,543 (35.2)** | **N/A** | **1,052 (20.6)** | **N/A** |
| ACEI / ARB / ARNI + beta blocker | 12,194 (31.7) | 12,194 (90.0) | 948 (18.5) | 948 (90.1) |
| ACEI / ARB / ARNI + MRA | 501 (1.3) | 501 (3.7) | 35 (0.7) | 35 (3.3) |
| ACEI / ARB / ARNI + SGLT2I | 277 (0.7) | 277 (2.0) | 19 (0.4) | 19 (1.8) |
| Beta blocker + MRA | 427 (1.1) | 427 (3.2) | 31 (0.6) | 31 (2.9) |
| Beta blocker + SGLT2I | 124 (0.3) | 124 (0.9) | 17 (0.3) | 17 (1.6) |
| MRA + SGLT2I | 20 (0.1) | 20 (0.1) | 2 (<0.1) | 2 (0.2) |
| **3-class** | **12,315 (32.0)** | **N/A** | **1,878 (36.7)** | **N/A** |
| ACEI / ARB / ARNI + beta blocker + MRA | 8,636 (22.4) | 8,636 (70.1) | 1,308 (25.6) | 1,308 (69.6) |
| ACEI / ARB / ARNI + beta blocker + SGLT2I | 3,337 (8.7) | 3,337 (27.1) | 515 (10.1) | 515 (27.4) |
| ACEI / ARB / ARNI + MRA + SGLT2I | 207 (0.5) | 207 (1.7) | 36 (0.7) | 36 (1.9) |
| MRA + beta blocker + SGLT2I | 135 (0.4) | 135 (1.1) | 19 (0.4) | 19 (1.0) |
| **4-class** | **8,405 (21.8)** | **N/A** | **2,039 (39.8)** | **N/A** |

ACEI = angiotensin-converting enzyme inhibitor; ARB = angiotensin receptor blocker; ARNI = angiotensin receptor-neprilysin inhibitor; CRT-D = cardiac resynchronization therapy defibrillator; GDMT = guideline-directed medical therapy; ICD = implantable cardiac defibrillator; MRA = mineralocorticoid receptor antagonist; SGLT2I = sodium glucose cotransporter-2 inhibitor.
^a^Device specifically refers to placement of an ICD/CRT-D device.
